# Non-Coding RNA: Role in Gestational Diabetes Pathophysiology and Complications

**DOI:** 10.3390/ijms21114020

**Published:** 2020-06-04

**Authors:** Tiziana Filardi, Giuseppina Catanzaro, Stefania Mardente, Alessandra Zicari, Carmela Santangelo, Andrea Lenzi, Susanna Morano, Elisabetta Ferretti

**Affiliations:** 1 Department of Experimental Medicine, “Sapienza” University, 00161 Rome, Italy; tiziana.filardi@uniroma1.it (T.F.); giuseppina.catanzaro@uniroma1.it (G.C.); stefania.mardente@uniroma1.it (S.M.); alessandra.zicari@uniroma1.it (A.Z.); andrea.lenzi@uniroma1.it (A.L.); susanna.morano@uniroma1.it (S.M.); 2 Center for Gender-Specific Medicine, Gender Specific Prevention and Health Unit, Istituto Superiore di Sanità, 00161 Rome, Italy; carmela.santangelo@iss.it

**Keywords:** non-coding RNA, microRNA, long non-coding RNA, circular RNA, gestational diabetes mellitus, GDM, GDM pathophysiology, pregnancy complications, placenta, epigenetics

## Abstract

Gestational Diabetes Mellitus (GDM) is defined as glucose intolerance that develops in the second or third trimester of pregnancy. GDM can lead to short-term and long-term complications both in the mother and in the offspring. Diagnosing and treating this condition is therefore of great importance to avoid poor pregnancy outcomes. There is increasing interest in finding new markers with potential diagnostic, prognostic and therapeutic utility in GDM. Non-coding RNAs (ncRNAs), including microRNAs, long non-coding RNAs and circular RNAs, are critically involved in metabolic processes and their dysregulated expression has been reported in several pathological contexts. The aberrant expression of several circulating or placenta-related ncRNAs has been linked to insulin resistance and β-cell dysfunction, the key pathophysiological features of GDM. Furthermore, significant associations between altered ncRNA profiles and GDM-related complications, such as macrosomia or trophoblast dysfunction, have been observed. Remarkably, the deregulation of ncRNAs, which might be linked to a detrimental intrauterine environment, can lead to changes in the expression of target genes in the offspring, possibly contributing to the development of long-term GDM-related complications, such as metabolic and cardiovascular diseases. In this review, all the recent findings on ncRNAs and GDM are summarized, particularly focusing on the molecular aspects and the pathophysiological implications of this complex relationship.

## 1. Introduction and GDM Overview

Gestational Diabetes Mellitus (GDM) is a metabolic disease diagnosed in the second or third trimester of pregnancy [1]. This condition is increasingly frequent worldwide due to the large spread of obesity [2]. Unless properly diagnosed and treated, GDM can lead to poor pregnancy outcomes. Women with GDM are at increased risk of gestational hypertension and pre-eclampsia [3]. Shoulder dystocia, birth trauma and preterm delivery, due to excessive foetal growth, are common birth complications [4]. Other short-term adverse outcomes include hypoglycaemia, hypoxia, risk of stillbirth and respiratory distress syndrome [5]. Remarkably, the negative impact of GDM is observed even later in life, both in the mother and in the offspring. Importantly, mothers previously diagnosed with GDM display an increased risk of developing type 2 diabetes (T2D) and metabolic syndrome [6,7]. Similarly, a higher risk of metabolic diseases and cardiovascular disease (CVD) has been reported in children born to mothers affected by GDM over adulthood [8,9]. 

Current diagnostic criteria for GDM have been established by the International Association of Diabetes and Pregnancy Study Groups (IADPSG). A strong link between high maternal glucose and the occurrence of adverse pregnancy outcomes has emerged in longitudinal studies, helping individuate the glycemic thresholds for GDM diagnosis [3]. According to the IADPSG recommendations, an oral glucose tolerance test (OGTT) with 75 g-glucose is performed at 24–28 weeks of gestation. Fasting, 1-h and 2-h plasma glucose are assessed and the cut-off values of glycaemia at OGTT are 5.1 mmol/L, 10 mmol/L and 8.5 mmol/L, respectively. One or more values exceeding or equaling these thresholds establish the diagnosis of GDM [10]. Although the IADPSG does not recommend routinely screening before 24–28 weeks of gestation, detecting GDM as early as possible is crucial to avoid poor pregnancy outcomes. Accordingly, a screening approach that takes into account the main GDM risk factors, such as obesity and previous GDM, might anticipate the diagnosis and prevent complications [11,12,13]. In addition, lifestyle, such as diet, physical activity or emerging environmental factors are likely to have an impact on the risk of developing GDM [13,14,15]. The identification of phenotypic classes associated with adverse outcomes, defined by the presence of some risk factors, turned out to be potentially useful in customizing and modulating the diagnostic and therapeutic approach in GDM [16,17]. Preventing and treating this condition likely contributes to limiting the burden of the disease. Accordingly, there is growing interest in identifying new markers with diagnostic, prognostic and therapeutic uses in GDM. Non-coding RNAs (ncRNAs) are important players in metabolic processes and their deregulated expression has been observed in several metabolic diseases, including GDM.

Insulin resistance and β-cell dysfunction are the main pathophysiological features of GDM. Insulin sensitivity significantly varies throughout pregnancy, constantly adapting to the energy demands of the mother and the foetus. Overall, insulin sensitivity follows a biphasic trend in healthy pregnancy, experiencing a sharp increase initially and a marked reduction as pregnancy evolves. The metabolic adaptation observed in the initial phase (the first two trimesters) is aimed at storing essential sources of energy, such as glucose and fatty acids, as fat deposits, necessary for the next stages of pregnancy [18]. Estrogens and progesterone synthesis progressively increases, together with other molecules of placental origin, such as human placental lactogen and human placental growth hormone, contributing to the progressive fall in insulin sensitivity. Thus, the insulin resistance state observed in physiological pregnancy is an adaptive response that favors the rise in glucose and free fatty acid blood levels, shifting energy sources from the mother to the foetus [19,20]. The pancreatic β-cell largely compensates through an increased insulin release. Hypertrophy and hyperplasia of β-cell mass, explained by enhanced proliferation and reduced apoptosis, have been reported in human pregnancy [21]. However, if β-cell dysfunction occurs, the compensative effect is lost and GDM becomes manifest. 

Insulin resistance is a consequence of impaired peripheral insulin signaling. Indeed, glucose uptake is almost halved and insulin resistance is enhanced in GDM compared to healthy pregnancy [22]. Insulin signaling is affected by altered phosphorylation of the insulin receptor or insulin receptor substrate (IRS)-1, although the number of receptors on the cell surface is preserved [23]. Remarkably, pro-inflammatory cytokines contribute to the development of insulin resistance [24,25]. Adipose tissue and gestational tissues are able to secrete pro-inflammatory cytokines, such as TNF-α, IL-1-β and IL-6, that impair insulin signaling by inhibiting IRS-1 through serine phosphorylation [26,27]. 

As regards β-cell dysfunction, it has been observed that β-cell function is reduced by 30–70% in GDM, indicating that β-cells are unable to compensate for the increase in insulin resistance, resulting in the development of GDM [28]. 

The mechanisms underlying β-cell dysfunction are not fully uncovered, but are likely to overlap those described in T2D. Largely, alterations at each step of insulin synthesis or secretion have been described, and β-cell dysfunction is triggered by hyperglycemia and hyperlipidemia [29]. Oxidative stress, mitochondrial dysfunction, endoplasmic reticulum stress are well-established consequences of glucotoxicity and lipotoxicity, and impair insulin synthesis, secretion and β-cell survival [30]. Pro-inflammatory cytokines further promote endoplasmic reticulum stress, and are able to induce β-cell de-differentiation [31,32]. 

The aim of this review is to report recent evidence about the role of ncRNAs in GDM. The understanding of their functions might help unravel the complex pathophysiological mechanisms in GDM and contextually identify novel potential diagnostic biomarkers for the disease.

## 2. General Aspects of NcRNAs

NcRNAs represent about the 60% of the transcriptional production of human genome and play central roles in pathways regulation, developmental and pathological processes [33,34,35,36]. 

On the basis of their length, ncRNAs are divided in two main categories: under 200 ribonucleotides are termed small ncRNAs, such as microRNAs, while over 200 ribonucleotides are named long ncRNAs (lncRNAs). Additionally, a new class of lncRNAs is represented by circular RNAs (circRNAs), derived by a process of “back-splicing” of mRNA sequences with the covalent binding between the 3′ and 5′ ends [37,38].

MicroRNAs are small non-coding single stranded RNAs of about 22 nucleotides that function as guide molecules in RNA silencing [39]. The latest miRbase version (version 22.1 released in October 2018) counts 2654 human mature microRNAs sequences [40]. Although the function of many of these microRNAs needs to be defined, the high number of discovered microRNAs and their wide distribution across species underline their pivotal role in gene regulation. Moreover, their dysregulation is involved in various diseases, spanning from cancer to metabolic diseases [41,42].

MicroRNAs act by pairing to their target mRNA sequence and can be encoded by intronic regions of coding or non-coding transcripts or, less frequently, by exonic regions [39]. RNA polymerase II and, less commonly, RNA polymerase III transcribe microRNA genes. The long primary sequence is capped at the 5′ and polyadenylated at the 3′ end. The derived pri-miRNA is recognized by the Microprocessor complex, constituted by the nuclear protein Di George Syndrome Critical Region 8 (DGCR8) and the RNAse III Drosha. This first nuclear maturation step releases a precursor microRNA, called pre-miRNA, translocated to the cytoplasm by the Exportin-5 protein. Into the cytoplasm the pre-miRNA is cleaved by the RNAse III Dicer, producing a double strand microRNA of about 22 nucleotides, that is loaded onto Ago2, a member of the Argonaute (Ago) protein family, to form a big ribonucleoprotein effector complex, termed RNA-induced silencing complex (RISC). The binding with Ago2 favors the most stable strand. The passenger strand can be degraded to produce a mature RISC or may sometimes act as another functional microRNA. In case of a complete sequence homology between the microRNA and its target mRNA, this can be cleaved by Ago proteins, with the final event of degradation of the target mRNA. Conversely, if there is no complete homology, the RISC complex can induce translational repression [43,44].

As microRNAs, also lncRNAs do not have the capacity for protein coding. However, differently from microRNAs, they are heterogeneous in size and share only few structural, functional and mechanistic features [37,45], being localized in badly conserved genomic regions [46,47]. Moreover, their expression is strictly regulated both in space and in time, therefore a dysregulated expression profile is an important marker of disease or of an altered developmental state. Even though a wide number of existing lncRNAs have been discovered, underlying their crucial role in numerous biological processes, their functional significance has been defined only for a small fraction so far. LncRNAs are transcribed by RNA polymerase II from intergenic, exonic or distal protein-coding regions of the genome and are usually 3′-polyadenylated, 5′-capped and spliced, generating three types of lncRNAs: competitor, recruiter or microRNA precursor, on the basis of their way of acting [48,49]. LncRNAs subcellular localization is a reliable hint of their modes of action: nuclear lncRNAs are primarily involved in the epigenetic gene regulation or in the maintenance of the nuclear architecture, while cytoplasmic lncRNAs are especially involved in post-transcriptional gene regulation. Interestingly, lncRNAs may function as gene regulators by acting as microRNA sponges and modulating microRNA post-transcriptional silencing [45,48]. 

Another interesting class of ncRNAs are circRNAs, lncRNAs that undergo a peculiar 5′- and 3′- processing with the production of a chemically circularized transcript that avoids exonucleolytic degradation by RNase R [50]. The circRNAs identified in human cells are more than 100,000 and can be generated through three different mechanisms that may require intron repetitive sequences either a circularization induced by RNA binding proteins (RBPs) or an exon skipping circularization, that produces the most studied type of circRNAs, called “ecircRNAs”. [38,49,51,52]. Since circRNAs may regulate gene expression at multiple levels, such as by sponging microRNAs or interacting with proteins, many studies started to evaluate their role in diseases, hypothesizing possible functions in pathological contexts with clinical implications [38,49].

MicroRNAs, such as lncRNAs and circRNAs, are produced and act into the cellular compartment. However, recent evidence demonstrated that they can be secreted and act into the extracellular compartment too. Although their function and origins are not yet fully understood, one of the most interesting and reliable hypothesis is that extracellular ncRNAs can mediate cell-to-cell communication, both in physiological and pathological contexts [37,53,54]. Recent studies demonstrated that they can be found in many biological fluids, including serum, plasma and urine. Specifically, ncRNAs can be actively secreted by cells after packaging into shedding vesicles and exosomes or coupling with protein complexes freely circulating in biological fluids. Otherwise they may be incorporated in apoptotic bodies, derived by apoptotic cells, and passively secreted [55].

Interestingly, circRNAs are enriched in extracellular vesicles compared to their levels in producer cells and their encapsulation into extracellular vesicles seems to be, at least partly, regulated by cellular microRNAs [56].

Furthermore, one of the most interesting properties of circulating ncRNAs is their stability into the extracellular environment: the association with protein complexes and/or the packing into extracellular vesicles protects them from RNAses activity, sudden temperature changes and extreme pH values, rendering them not only very stable as extracellular molecules, but also capable to act as diagnostic, prognostic and therapeutic biomarkers [57,58,59,60,61]. 

## 3. NcRNAs: Insights into the Pathophysiology of GDM

### 3.1. Insulin Resistance and Metabolic Adaptations

Placenta and placenta-derived molecules play an essential role in pregnancy and in the pathogenesis of GDM. Many studies addressed the role of placenta-derived microRNAs in healthy and GDM pregnancies [62,63]. Interestingly, placenta-associated microRNAs have been described as major regulators of peroxisome proliferator-activated receptors (PPARs). PPARs are a family of fatty acid receptors with critical involvement in energy control, lipid homeostasis and inflammation and have been already linked to T2D [64]. In 2014, Zhao et al. demonstrated a negative correlation between the level of expression of miR-518d and PPARα. MiR-518d, a placenta specific microRNA [65], belongs to the C19 MC cluster and has a higher expression at 37–40 weeks of gestation in placentas derived from n = 40 women affected by GDM in respect to n = 40 healthy pregnant women. They further demonstrated the direct control of miR-518d over the expression of PPARα [66]. A more recent study investigated the expression and role of miR-143 in placental tissues from pharmacologically treated GDM patients (n = 6), GDM patients treated only with diet (n = 6) and women with uncomplicated pregnancy (CTRL, n = 6). Mir-143 was involved in the dysregulation of the mitochondrial function observed in placentas from pharmacologically treated patients. Specifically, Muralimanoharan et al. demonstrated that while miR-143 was significantly downregulated in pharmacologically-treated patients in respect to diet-treated patients and CTRL women, the glycolytic enzymes, GLUT1, HK-2, PFK and LDH, were significantly upregulated. Moreover, they showed that miR-143 directly regulated HK-2, the first enzyme of the glycolytic pathway, and that miR-143 overexpression in trophoblast cells derived from pharmacologically treated patients was able to partially rescue mitochondrial function and reduce glycolytic enzymes [67]. 

Another interesting microRNA that is involved in T2D and in embryo implantation at the beginning of pregnancy is miR-98 [68,69,70]. In 2016, Cao et al. demonstrated the involvement of miR-98 in GDM. Specifically, they showed that miR-98 expression was significantly higher in placental tissue recovered at 37–40 weeks from GDM pregnant women (n = 193) than in normal pregnant women (n = 202). Moreover, by using the human choriocarcinoma cell line JEG-3, they demonstrated that miR-98 not only induced DNA methylation but also directly regulated Mecp2, which in turn targeted Trpc3, a major regulator of insulin-mediated glucose uptake. These data confirm the role of miR-98 in the regulation of glucose uptake in GDM [71].

The placenta has been widely demonstrated to promote the metabolic adaptations in pregnancy, critically contributing to the onset of insulin resistance. Interestingly, it is believed that the metabolic status of the placenta might be mirrored by the microRNA expression profile in placenta-derived exosomes. Nair et al. explored the exosome microRNA expression by next generation sequencing (NGS) in chorionic villi explants from 12 women with GDM and 12 CTRLs [72]. Overall, 13 microRNAs were significantly upregulated, while 14 microRNAs were downregulated in GDM compared to CTRLs. The authors further evaluated the expression of selected microRNAs (miR-22-3p, miR-125a-3p, miR-197-3p, miR-99b-5p, and miR-224-5p) in skeletal muscle biopsies and in circulating exosomes, highlighting different patterns of expression between GDM and CTRLs, which broadly reflected the chorionic villi profile. Of note, the predicted target genes of the deregulated microRNAs were largely involved in glucose metabolism. Specifically, the phosphatidylinositol 3-kinase (PI3K)/protein kinase B (AKT) signaling pathway was targeted by several differentially expressed microRNAs. It is well known that the activation of PI3K/AKT pathway by the insulin tyrosine kinase receptor promotes glucose uptake, inducing the translocation of glucose transporter 4 (GLUT4) on the cell surface [73]. Hence, placenta-derived exosomes might play a role in the cross-talk between gestational tissues and skeletal muscle, possibly modulating peripheral insulin action in GDM [63]. 

High insulin levels in a context of insulin resistance are thought to modulate circulating microRNA expression. Stirm et al. observed significantly higher levels of miR-340 in whole blood cells (WBC) and in lymphocytes in patients with GDM (n = 8) than in CTRLs (n = 8) at 24–32 weeks of pregnancy [74]. The mRNA of polyadenylate (poly(A))-binding protein (PABP)-interacting protein 1 (PAIP1) displayed significant downregulation in WBCs, but not in lymphocytes, in patients with GDM, compared to normal glucose tolerant (NGT) women. However, in lymphocytes, an inverse correlation between miR-340 and PAIP1 protein expression was detected, suggesting that PAIP1 expression might be regulated by miR-340. PAIP1 is a key promoter of translation, mainly studied in cancer contexts and never evaluated before in GDM [75]. Human lymphocytes were cultured in high glucose, reproducing in vitro fasting and after meal glycaemia. High glucose reduced miR-340 levels. The addition of insulin to high glucose medium, reflecting a condition of high fasting glycaemia and insulin resistance, led to a significant increase in miR-340 levels, compared to cells cultured without insulin, indicating a critical effect of insulin on miR-340 expression. Accordingly, in vivo maternal fasting insulin levels were positively associated with miR-340 expression in WBCs, indicating that high fasting insulin, observed in insulin resistance states, might modulate microRNA expression in WBCs.

MicroRNA activity is critically regulated by circRNAs, acting as miRNA sponges for the modulation of their target genes. The expression of circRNAs in placental villi has been explored in three GDM and in three NGT women [76]. A large number of circRNAs turned out to be differentially expressed in GDM and their predicted target genes were involved in glucose and lipid metabolism. The insulin, the glucagon, the adenosine-monophosphate (AMP)-activated protein kinase (AMPK) and the cytokine signaling pathways, crucial contributors in the pathophysiology of GDM, were targeted. Specifically, AMPK has been found to phosphorylate key proteins and transcription factors, modulating glucose and lipid metabolism. In particular, glucose and fatty acid uptake are promoted by AMPK in the skeletal muscle and in the liver. Mitochondrial function and fatty acid oxidation are enhanced, whereas lipid and cholesterol synthesis are suppressed. Furthermore, it has been observed that AMPK inhibits the pro-inflammatory NF-κB signaling pathway, one of the main modulators of gene expression in pro-inflammatory responses. Notably, AMPK activity was reported to be significantly reduced in adipose tissue and in skeletal muscle in women with GDM [77,78,79]. Furthermore, Muralimanoharan described a downregulation of pAMPK in placenta from pharmacologically treated GDM patients, that could be responsible for mTOR pathway activation and could be involved in the shift toward aerobic glycolysis observed in GDM [67].

Less is known about the role of adipose tissue derived microRNAs in the pathogenesis of GDM. Omental adipose tissue is associated with a more adverse metabolic risk profile than subcutaneous adipose tissue [80] and could be involved in the development of insulin resistance [81]. To our knowledge, there is only one report that comparatively evaluated the microRNA expression profile in omental adipose tissues from GDM (n = 3) and healthy pregnant women (n = 3) at caesarean delivery. By using Affymetrix microRNA expression chips, Shi et al. identified 17 differentially expressed microRNAs. However, they focused their attention only on the upregulated miR-222 in order to study the relationship between its expression and insulin resistance in GDM. Specifically, in the validation study, conducted on ten GDM and ten healthy pregnant women, they showed a negative correlation between miR-222 levels, estrogen receptor (ER)-α and GLUT4 and an increase in serum estradiol level in GDM women in respect to CTRLs. This correlation and the direct targeting of miR-222 on ER-α were functionally validated in 3T3-L1 adipocytes. Since estradiol and ER-α, acting on GLUT4, are critical regulators in obesity and insulin resistance [82,83,84] these results underline the role of miR-222 in the modulation of ER-α expression in estrogen-induced GDM insulin resistance.

### 3.2. β-Cell Dysfunction 

Maternal pancreatic β-cell dysfunction is critically involved in the pathogenesis of GDM. Only a few studies have evaluated the relationship between ncRNAs and β-cell dysfunction in GDM so far. Although still limited, evidence indicates a conceivable ncRNA-mediated cross-talk between placenta and β-cells in GDM, enriching the complex pathophysiological picture in GDM.

The whole transcriptome expression has been profiled by high-throughput sequencing in placentas from three women with GDM and three NGT, after caesarean section [85]. A large number of ncRNAs (microRNAs, lncRNAs and circRNAs) were found to be upregulated or downregulated in GDM compared to healthy pregnancy. Further validation by qRT-PCR confirmed the sequencing results. Of note, the main predicted target genes of the differentially expressed ncRNAs were linked to molecular pathways known to critically regulate glycolipid metabolism and β-cell function, such as the sphingolipid [86], the Notch [87], the thyroid hormone receptor [88] and the prolactin signaling pathways [89]. In particular, the sphingolipid signaling pathway is involved in the development of lipotoxicity [86], regulating ceramide metabolism in peripheral tissues (skeletal muscle, liver and adipose tissue) and in pancreatic β-cells. The accumulation of ceramides seems to play a role in the development of insulin resistance and β-cell dysfunction [86]. As regards prolactin signaling, it has been observed that its deletion in β-cells slows proliferation, therefore contrasting β-cell expansion, a crucial compensation mechanism for peripheral insulin resistance in pregnancy [89].

Similarly, Li et al. evaluated microRNA expression in placental tissue from three GDM and three CTRLs [90]. A significant downregulation of miR-96 was reported in GDM compared to CTRLs and an inverse correlation between miR-96 expression and blood glucose emerged. Notably, under high glucose condition the knockdown of miR-96 lowered insulin concentration and cell viability, and increased apoptosis in INS-1 cells. PAK1 was found to be a direct target of miR-96. While miR-96 enhanced β-cell function, PAK1 had opposite effects. Accordingly, a reduced β-cells mass was observed in PAK1-deficient mice, compared to control mice [91]. These results suggest that miR-96 downregulation might contribute to the impairment of β-cell function, by targeting PAK1. 

Sebastiani et al. assessed microRNA expression in plasma samples from patients with GDM (n = 21) and normal glucose tolerant women (n = 10) at 24–33 weeks of pregnancy [92]. A significant upregulation of miR-330-3p was found in GDM compared to CTRLs. In addition, within the GDM group, the authors identified two age and BMI matched subgroups, based on miR-330-3p expression levels. Interestingly, fasting insulin was lower in GDM with high miR-330-3p expression than in GDM with low miR-330-3p expression, and exhibited significant inverse correlation with circulating miR-330-3p levels only in GDM patients. A higher rate of caesarean section, related to pregnancy complications (fetal macrosomia and polyhydramnios), was observed in the high miR-330-3p group compared to the low miR-330-3p group, indicating that high miR-330-3p levels might label a more aggressive GDM phenotype, with increased risk of adverse pregnancy outcomes. E2F transcription factor 1 (E2F1) and Cell Division Cycle 42 (CDC42), known to be critical modulators of β-cell growth and proliferation, were validated as target genes of miR-330-3p, being downregulated by miR-330-3p over-expression. Notably, besides β-cell maintenance, E2F1 and CDC42 have been also shown to regulate glucose-stimulated insulin secretion [93,94]. Remarkably, PAK1, another well-established key regulator of β-cell mass and function, was involved in the predicted interaction network analysis. 

An interesting preclinical study revealed important regulating aspects of β-cell function in GDM [95]. The authors demonstrated the role of miR-410 in alleviating β-cell dysfunction in the db/+ mouse model of GDM. Specifically, Mi et al. transfected the H1 human embryonic stem cell (hESCs) line with miR-410, demonstrating its direct targeting of the lactate dehydrogenase A (LDHA), normally repressed in β-cells. Conversely, the hyper-expression of LDHA has been reported to inhibit glucose-stimulated insulin secretion [96]. The hESCs expressing miR-410 were then differentiated in vitro into pancreatic endoderm (PE) and transplanted into db/+ female mice. The female mice were followed for their levels of blood glucose, plasma insulin and body weight before and 4 weeks after the transplant, as well as during pregnancy (at the beginning, at 10 days and at 20 days of gestation). They demonstrated that miR-410 transplantation not only was able to improve β-cell function, promoting insulin secretion and, consequently, reducing plasma glucose levels, but also significantly improved pregnancy outcomes (reduction in miscarriage rate and foetal overgrowth, increased survival at birth) in GDM pregnant mice [95].

## 4. NcRNAs as Candidate Circulating Biomarkers of GDM 

Several studies have investigated the potential role of ncRNAs as circulating plasma/serum biomarkers for GDM (Table 1). 

In 2015, Zhu et al. conducted a pilot prospective study by collecting peripheral blood samples from pregnant women at 16–19 weeks, that were subsequently diagnosed or not with GDM, and analysing plasma microRNAs with high-throughput sequencing. They sequenced 187 microRNAs in women that later developed GDM (n = 10) and 156 microRNAs in the control group (n = 10) identifying 32 differentially expressed microRNAs. Five of them (miR-16-5p, miR-17-5p, miR-19a-3p, miR-19b-3p and miR-20a-5p), chosen on the basis of reads number and fold change, were upregulated in women that developed GDM. These microRNAs, further validated by means of RT-qPCR, were associated with insulin secretion, providing a microRNA aberrant signature that might predict GDM at an early stage of pregnancy [97]. All these five microRNAs were further investigated in plasma collected from n = 157 pregnant subjects, at first prenatal examination and then every 4 weeks until testing for GDM. Among them, n = 85 women developed GDM and n = 72 were NGT at 24–28 weeks of pregnancy. This analysis confirmed the significant upregulation of three microRNAs, namely miR-16-5p, miR-17-5p and miR-20a-5p, in pregnant women that developed GDM and a positive correlation with insulin resistance [98]. Furthermore, Wander et al. investigated the role of microRNAs as predictors of GDM by selecting ten candidate biomarkers on the basis of their involvement in pregnancy complications or pathophysiological pathway related to pregnancy complications. MicroRNAs were analysed on plasma derived from peripheral blood samples collected within 16 weeks of pregnancy from a sub-cohort of n = 116 women, enrolled in a larger pregnancy cohort study. The sub-cohort included n = 36 women that developed GDM at 24-28 weeks of pregnancy and n = 80 healthy subjects. Additionally, pregnant women were classified as overweight/obese according to pre-pregnancy body mass index (BMI). Only few microRNAs were associated with increased risk of GDM. Specifically, two microRNAs (miR-21-3p and miR-210-3p) were associated with a higher risk of GDM only in women who were overweight/obese prior to pregnancy, while several microRNAs (miR-155-5p, miR-21-3p, miR-146b-5p, miR-223-3p, miR-517-5p, miR-29-3p) were associated with an increased risk of GDM only in mothers bearing male offspring [99].

Other authors observed significantly reduced levels of miR-21-3p in blood leukocytes in GDM patients compared to NGT women at third trimester of pregnancy [100]. MiR-21-3p significantly predicted GDM, although with rather low sensitivity. Evidence suggests that miR-21-3p is involved in the mechanisms of β-cell compensation in response to insulin resistance in GDM [92].

Tagoma et al. explored microRNA expression profile in plasma samples obtained from women with GDM (n = 13) and without GDM (n = 9) at 25–27 weeks of pregnancy [101]. Several microRNAs were overexpressed in the GDM group compared to CTRLs, with miR-195-5p displaying the highest level of upregulation. Evidence suggests that the predicted target genes of miR-195-5p are involved in fatty acid synthesis and gluconeogenesis [102]. Notably, miR-195-5p upregulation has been linked to the development of insulin resistance [103]. 

Furthermore, Tagoma et al. observed an upregulation of miR-222-3p in women with GDM compared to healthy subjects. By contrast, Zhao et al. reported the opposite result [104]. However, Zhao et al. explored microRNA expression in serum samples at a different stage of pregnancy (16–19 weeks), when the metabolic adaptations are still not completely defined. In line with Zhao et al., a significantly decreased expression of miR-222-3p in sera of GDM patients (n = 53) compared to healthy pregnant women (n = 28) was reported by Pheiffer et al. [105]. A decreased expression of miR-20a-5p also emerged, and a role of miR-20a-5p as predictor of GDM, after adjustment for miR-222-3p levels, age, BMI and risk factors for GDM was postulated. 

Yoffe et al. detected the circulating microRNA profile in plasma samples in women with GDM and in healthy pregnant subjects at first trimester [106]. MiR-223 and miR-23a were significantly upregulated in GDM. Notably, in logistic regression analysis both microRNAs were significant predictors of GDM, indicating that miR-223 and miR-23a are potential biomarkers for GDM in the first trimester.

It should be highlighted that the contrasting results and the inconsistencies among studies might be explained by critical differences in many factors, such as the timing of sample collection, the biological fluid analysed (serum/plasma), the ethnicity of the enrolled population. Furthermore, not all studies have considered the effect of potential confounders, such as age and BMI. 

**Table 1:** Studies that evaluated circulating miRNAs in GDM.

Study	Groups	Stage of Pregnancy	Source	miRNA
Cao et al. [98]	85 GDM	16–28 weeks	Plasma	miR-16-5p, miR-17-5p, miR-20a-5p (↑)
	72 CTRL			
Lamadrid-Romero et al. [107]	13 GDM	1st trimester	Serum	1st trimester:
	12 CTRL			miR-125b-5p, miR-183-5p, miR-200b-3p, miR-1290 (↑)
	24 GDM	2nd trimester		2nd trimester:
	24 CTRL			miR-183-5p, miR-128-5p (↑)
				miR-125b-5p in the (↓)
	20 GDM	3rd trimester		3rd trimester:
				miR-137 (↑)
				miR-183-5p, miR-200b-3p (↓)
Wander et al. [99]	36 GDM	7–23 weeks	Plasma	miR-155-5p, miR-21-3p, miR-210-3p, miR-146b-5p, miR-223-3p, miR-517-5p, miR-29a-3p (↑)
	80 CTRL			
Zhu et al. [97]	10 GDM	16–19 weeks	Plasma	miR-16-5p, miR-17-5p, miR-19a-3p, miR-19b-3p, miR-20a-5p (↑)
	10 CTRL			
Tagoma et al. [101]	13 GDM	25–27 weeks	Plasma	miR-195-5p, miR-222-3p (↑)
	9 CTRL			
Sebastiani et al. [92]	21 GDM	24–33 weeks	Plasma	miR-330-3p (↑)
	10 CTRL			
Zhao et al. [104]	24 GDM	16–19 weeks	Serum	miR-29a, miR-123, miR-222 (↓)
	24 CTRL			
Pheiffer et al [105]	53 GDM	26–27 weeks	Serum	miR-222-3p, miR-20a-5p (↓)
	28 CTRL			
Yoffe et al. [106]	23 GDM	9–11 weeks	Plasma	miR-223, miR-23a (↑)
	20 CTRL			

GDM: gestational diabetes mellitus; CTRL: control subjects.

Evidence about the biological functions and role of lncRNAs as potential biomarkers of GDM are still limited (Table 2). Zhang et al. explored the potential relationship between three lncRNAs, namely lncRNA MALAT1, lncRNA H19, and lncRNA p21, and GDM. They analysed serum samples obtained between the 36th and the 40th week of pregnancy from n = 50 pregnant women affected by GDM and n = 47 healthy pregnant women. They demonstrated a higher expression of the lncRNA MALAT1 in GDM pregnant women, indicating a potential role of the lncRNA MALAT1, previously reported to be involved in the induction of diabetic microangiopathy [108], as a serum biomarker of GDM [109].

## 5. NcRNAs and GDM Complications

### 5.1. Short-Term Complications

Numerous studies have revealed a conceivable link between several dysregulated ncRNAs and impairments in placenta development and functions in GDM. 

Ding et al. uncovered a set of deregulated microRNAs by RNA sequencing and further qRT-PCR validation in placental tissues from eight GDM women compared to eight CTRLs [114]. An integrative evaluation of microRNA and mRNA expression was performed and the predicted biological functions of the differentially expressed microRNA and mRNA were related to placenta morphology and development. The authors selected miR-138-5p, which was significantly overexpressed in GDM, for functional assays. Notably, the hyper-expression of miR-138-5p inhibited the migration ability and the proliferative activity of HTR-8/SVneo trophoblast cells. MiR-138-5p targeted TBL1X, an oncogene that activates WNT/β-catenin signaling, a key pathway, crucially involved in numerous placental physiological processes, such as proliferation, differentiation and invasion [115,116].

Other authors revealed an association between the upregulation of miR-137 and hallmarks of trophoblast dysfunction, possibly contributing to the development of adverse outcomes in GDM [117]. Protein kinase AMP-activated catalytic subunit α1 (PRKAA1) was decreased in placental tissues from women with GDM (n = 11) compared to CTRLs (n = 11). In vitro, high glucose upregulated miR-137 and IL-6 in HTR-8/SVneo trophoblast cells and the overexpression of miR-137 decreased the levels of PRKAA1, while increased IL-6. Overall, these modifications impaired the viability and the proliferation activity of HTR-8/SVneo trophoblast cells.

Wang et al. investigated the biological function of the lncRNA plasmacytoma variant translocation 1 (PVT1) in placental tissues from patients with GDM (n = 13), patients with pre-eclampsia (n = 15) and healthy pregnant women (n = 13) [110]. A lower expression of PVT1 was observed in GDM and in pre-eclampsia group, compared to normal pregnancy. In functional assays, the knockdown of PVT1 had detrimental effects on proliferation and migration of HTR-8/SVneo trophoblast cells. A decrease in AKT phosphorylation also emerged. Notably, PVT1 regulates a large number of microRNAs, therefore modulating the expression of their target genes in trophoblast cells. Bioinformatics analysis predicted several microRNAs (miR-10-5p, miR-423-5p, miR-374b-5p, miR-378b, miR-150-5p, miR-194-5p and miR-3184) that likely target the PI3K/AKT pathway.

Several ncRNAs have been linked to the development of endothelial dysfunction (ED) in pregnancy complicated by GDM, possibly contributing to poor pregnancy outcomes. Peng et al. observed significantly increased plasma levels of miR-137 in GDM (n = 11), compared to healthy pregnant women (n = 12) at third trimester [118]. Accordingly, other authors consistently reported higher expression of miR-137 in peripheral blood and placental tissue in patients with GDM, compared to CTRLs [56,97,104]. Remarkably, miR-137 expression significantly rose in human umbilical vein endothelial cells (HUVECs) in vitro after exposure to high glucose. The increased expression of miR-137 in HUVECs was associated with impaired viability and angiogenesis. Contextually, a higher expression of superficial markers, such as vascular cell adhesion molecule-1 (VCAM-1), intercellular cell adhesion molecule-1 (ICAM-1) and E-selectin was detected, together with increased interleukin IL-6 and reduced IL-8 expression [118]. Hence, a role of miR-137 as promoter of ED in GDM might be speculated. Another study focused on the role of microRNA dysregulation in GDM in the context of ED. In HUVEC cells derived from n = 22 GDM and n = 24 healthy pregnant women, Floris et al. demonstrated that miR-101 level, whose role in controlling endothelial function and angiogenesis is well described [119], was higher in GDM derived HUVECs (GDM-HUVECs) than in healthy HUVECs. One of the main miR-101 targets is the histone methyltransferase enhancer of zester homolog 2 (EZH2). EZH2 regulates angiogenesis [119,120,121,122,123,124], and Floris et al. demonstrated not only a reduction in its β-isoform in GDM-HUVECs, but also a feedback loop between EZH2 and miR-101. Specifically, the inhibition of miR-101 decreases EZH2 levels in GDM-HUVECs and the silencing of EZH2 in GDM-HUVECs or the overexpression of EZH2- β in healthy HUVECs induced the increase in miR-101 expression. These results indicate a reciprocal regulation of miR-101 and EZH2 in HUVECs and suggest that the imbalance observed in GDM might be responsible for the higher expression of miR-101 and the functional alterations of endothelial cells, such as decreased survival and functional capacity [125]. 

Mounting evidence therefore indicates that foetal endothelial function is considerably impaired in GDM pregnancies. Ye et al. reported a fall in proliferation and a rise in apoptosis, as well as impaired tube formation and migration, in HUVECs obtained from GDM patients compared to healthy women, under high glucose exposure [111]. Furthermore, a higher expression of the lncRNA maternally-expressed gene 3 (MEG3) emerged in HUVECs from GDM compared to CTRLs. MEG3 overexpression downregulated miR-370-3p and inhibited the PI3K/AKT pathway, which is thought to be involved in hyperglycaemia-induced ED [126]. 

NcRNAs may also play important roles in regulating foetal growth during pregnancy [127,128]. Since one of the main complications of GDM is foetal macrosomia, the identification of dysregulated ncRNAs that may be involved in this phenomenon could be of utmost importance. In 2015, two studies addressed a link between ncRNAs and foetal macrosomia. Li et al. conducted a microarray analysis to compare the microRNA profiles of placenta derived from healthy (n = 15) and GDM (n = 15) pregnant women immediately after delivery. Interestingly, they identified miR-508-3p as the most upregulated microRNA in GDM samples, and demonstrated its direct regulation of PIKfyve, which was concordantly significantly downregulated. PIKfyve is a FYVE-containing phosphoinositide 3-phosphate-5-kinase that acts as a negative regulator of the epidermal growth factor receptor (EGFR) [129]. Thus, the upregulation of miR-508-3p in GDM women contributes to the repression of PIKfyve and the aberrant activation of the EGFR/PI3K/AKT signalling, contributing to foetal overgrowth [56]. 

Shi et al. evaluated the association between lncRNAs and macrosomia. They analysed the lncRNA expression profile in plasma from umbilical cord vein in pregnancies with GDM-induced macrosomia (n = 30) and with non-diabetic macrosomia (n = 30). In particular, they identified 349 significantly upregulated lncRNAs and 892 significantly downregulated lncRNAs in the GDM-induced macrosomia group, compared to the non-diabetic macrosomia group. They chose to validate only eight of these differentially expressed ncRNAs, on the basis of the relationship of their target genes with foetal growth, glucose and lipid metabolism. Interestingly, ENST00000552367, the most upregulated lncRNA in the GDM group, targeted the T cell death-associated gene 51 (TADG51), which is involved in the anti-apoptotic effect of insulin-like growth factor-1, an important regulator of cell proliferation and GDM-induced macrosomia [112]. 

Lu et al. analyzed the lncRNA expression profile through microarray at an early stage of gestation, in plasma samples from GDM patients with fetal macrosomia (n = 3), GDM patients without fetal macrosomia (n = 3), pregnant healthy women (n = 3) and non-pregnant healthy controls (n = 3) [113]. Five lncRNAs (XLOC_014172, RP11-230G5.2, PCBP1-AS1, LOC149086 and RP11-160H22.5) resulted to be associated with macrosomia in pregnancy. However, only XLOC_014172 and RP11-230G5.2 were further validated in larger samples (150 subjects each group) and displayed moderate ability to predict macrosomia in GDM pregnancy. 

Mughal et al. evaluated the effect of GDM on myogenesis. They described a signaling network that involves miR-133a, a microRNA expressed in all muscle lineages, implicated in muscle growth and mitochondrial function regulation [130,131,132], together with the diacylglycerol-protein kinase C (PKC) δ and the myocyte enhancer factor-2 (MEF2), involved in the development of mitochondrial dysfunction in GDM. Specifically, in the presence of lipotoxicity, the repression exerted by MEF2 on miR-133a is responsible for the increase in the expression, and subsequent activity, of Nix, a miR-133a target belonging to the BCL-2 family, leading to the regulation of mitochondrial function in muscle tissues [133]. Moreover, Nix and miR-133a might be involved in the development of cardiac hypertrophy and diabetic cardiomyopathy [134,135,136], with a possible important role in heart failure progression following cardiac injury [133]. 

Lamadrid-Romero et al. instead analyzed the modulation of fetal neural development related to serum microRNA expression over the three trimesters of pregnancy in GDM patients (n = 13, n = 24, n = 20 at first, second and third trimester, respectively) and in CTRLs (n = 12, n = 24, n = 16 at first, second and third trimester, respectively) with the aim to find reliable biomarkers for fetal central nervous system (CNS) development. They also included a small cohort of non-pregnant women (Np, n = 10), since only microRNAs expressed at very low level, or even absent, in Np may be considered as possible biomarkers for the development of the fetal CNS. On this basis, they chose 12 microRNAs related to neural development and observed that GDM affected their levels differently in each trimester. Among them, miR-183-5p, miR-200b-3p, miR-125b-5p and miR-1290 were upregulated in GDM patients in respect to CTRLs in the first trimester of pregnancy. The number of differentially expressed microRNAs was reduced during the second and the third trimester. Specifically, during the second trimester, miR-183-5p was higher and miR-128-5p was lower in the GDM group in respect to control group. In the third trimester miR-137 was higher, while miR-183-5p and miR-200b-3p were lower in the GDM group. These differences underline that microRNAs might exhibit temporary regulation during pregnancy and that GDM affects microRNA expression pattern with subsequent possible effects on cell proliferation and neurogenesis, especially during the first trimester [107].

### 5.2. Long-Term Complications

Mounting evidence suggests that gene expression in the offspring might be altered by an adverse intrauterine environment, even in the absence of underlying changes in DNA sequences, and the variations in microRNA expression are believed to mediate this process [137]. 

MicroRNAs are crucial regulators of endothelial function. ED is suspected to play a role in the development of long-term GDM-related complications, such as metabolic diseases and CVD, both in the mother and in the offspring. The imbalance between vasodilator and vasoconstrictor factors, as well as the enhanced expression of adhesion molecules and growth factors, contribute to extensive endothelial injury, therefore promoting the onset of atherosclerosis [73,138,139]. ED is further worsened by the pro-inflammatory state that weights on GDM complicated pregnancy [140]. 

Strutz et al. isolated primary human arterial feto-placental endothelial cells (fpEC) from the placenta of GDM (n = 14) and healthy pregnant women (n = 14) to explore microRNA expression profile by NGS [141]. As the feto-placental endothelium is directly connected with foetal circulation, fpEC might reflect the metabolic status in foetal environment. GDM significantly regulated 26 microRNAs, compared to normal pregnancy. Remarkably, critical differences in microRNA expression emerged when stratifying by foetal sex. Specifically, only four microRNAs were altered by GDM in fpEC from male foetuses, whereas 22 microRNAs were differentially expressed in fpEC from female foetuses, suggesting that the modifications in microRNA profile in GDM might be significantly addressed by foetal gender. Interestingly, microRNA deregulation in GDM was more marked in female sex than in male sex, suggesting that female-related fpECs are possibly more prone to develop metabolic alterations in GDM.

Diaz-Perez et al. demonstrated a low expression level of both the cell adhesion molecule ICAM-1 in primary human fpEC and its soluble form in the supernatant of fpEC derived from four GDM women in respect to four CTRLs. Since miR-221 and miR-222 are two negative regulators of ICAM-1 [142,143] and are significantly upregulated in fpEC cells derived from GDM in respect to normal pregnancies, Diaz-Perez inferred that these microRNAs may determine the reduced ICAM-1 levels observed in the feto-placental endothelium in GDM [144]. Another study compared the microRNA expression in HUVECs derived from three infants from GDM women (IGDM) and from three infants from normoglycaemic CTRLs. By using microarray analyses, followed by RT-qPCR validation studies, Tryggestad et al. demonstrated that seven microRNAs (miR-30c-5p, miR-452-5p, miR-126-3p, miR-130b-3p, miR-148a-3p, miR-let-7a-5p and miR-let-7g-5p) were upregulated in IGDM HUVECs in respect to CTRLs. They focused on the microRNAs whose targets were involved in energy metabolism, endothelial function and adipogenesis, and identified reduced levels of AMPKα1 and pAMPKα1, known to be involved in the stimulation of glucose uptake and fatty acid oxidation in placentae from both male and female IGDM offspring. In particular, they suggested that AMPKα1 decrease may intervene in the fat oxidation reduction described in 1-month infants, and may also predispose the offspring to future metabolic diseases [145].

Recently, microRNA expression was explored in skeletal muscle biopsies in the offspring (age 25–35 years) of mothers with previous GDM (n = 82), type 1 diabetes (n = 67) and healthy women (n = 57) [146]. MiR-15a and miRNA-15b levels were significantly higher in skeletal muscle in offspring from GDM mothers, compared to CTRLs, after adjustment for confounders. Overall, both microRNAs were positively associated with offspring fasting glucose, 2-h post-load glycaemia and HbA1c. Moreover, miR-15a levels showed significant positive correlation with maternal 2-h post-load glycaemia during pregnancy, independently of confounders, suggesting that hyperglycaemia and microRNA deregulation in pregnancy might contribute to the intrauterine fetal programming for metabolic disease later in life. It has been observed that miR-15b downregulates the insulin receptor [147] and impairs insulin signaling through an upregulation of the PI3K regulatory subunit 1 [148]. On the other hand, miR-15a intervenes in the regulation of insulin synthesis and secretion. Remarkably, chronic exposure to high glucose induced the downregulation of miRNA-15a, impairing insulin release in mouse β-cells [149].

A downregulation of miR-181a in umbilical-cord blood cells of newborns of GDM patients compared to healthy pregnancies has been described [150]. It is well established that miR-181a is involved in the modulation of insulin sensitivity and mitochondrial function in the liver, by regulating the target gene BCL2 [151]. Accordingly, a downregulation of BCL2 both in umbilical-cord blood cells of newborns from GDM and in peripheral blood cells in obese adults has been reported, suggesting that GDM neonates and obese subjects might share the same transcriptional alteration in BCL2 [150]. In this view, hyperglycemia in utero might promote transcriptional modifications that predispose the offspring to the development of metabolic diseases later in life.

Finally, Fornes et al. evaluated the effect of metabolic alterations in GDM rats on both male and female fetuses by analysing the fetal liver, a crucial organ in lipid metabolism regulation, after 21 days of pregnancy. Interestingly, they demonstrated that GDM differently affects liver growth on the basis of the fetal sex. Since PPARs are critical in the control of lipid metabolism, they focused their analysis on PPARs and proposed that sex-dependent changes of microRNAs in GDM rats during pregnancy are involved in PPARs regulation. Specifically, miR-130, which targets PPARγ, was upregulated only in the liver of male fetuses from GDM rats, where PPARγ was downregulated. Conversely, miR-122 was downregulated, while its target PPARδ was upregulated. Interestingly, PPARδ level was increased also in the liver of female fetuses from GDM rats, but in this case, it seemed to be under the control of miR-9, that was downregulated. These results underline that early sex-dependent differences in microRNAs could be able to determine different responses and adaptations to challenges in the life of the offspring [152].

## 6. Conclusions and Future Perspectives

GDM is an increasingly common condition that can lead to severe and lifelong adverse complications for the mother and the child. The pathophysiological features of GDM have been revealed and mainly include maternal insulin resistance, placental dysfunction, ED and inflammation. However, the molecular mechanisms involved in the pathophysiology of this condition, as well as in the development of its complications, are not yet fully defined. There is great interest in the potential roles of ncRNAs as regulators of biological processes, mediators of tissue cross-talk and biomarkers of disease. Thus, a growing number of studies have characterized ncRNA expression in biological fluids and in gestational tissues, highlighting their crucial involvement in the pathogenic mechanisms of GDM, such as insulin resistance and β-cell dysfunction, and GDM-related complications, such as trophoblast dysfunction and fetal macrosomia. 

It is well established that early diagnosis and appropriate treatment are essential elements to prevent poor pregnancy outcomes in GDM. According to current guidelines, GDM diagnosis is generally performed in the late second trimester, when the metabolic alterations have already developed and emerge at the OGTT. The identification of circulating biomarkers that might effectively predict GDM at an earlier stage of pregnancy is therefore crucial to avoid mother and fetal complications through prompt lifestyle and diet intervention. Evidence has suggested a potential function of several circulating ncRNAs as early predictors of GDM. The remarkable stability in extracellular fluids and the easy collection from peripheral blood samples are the main strengths of these molecules as putative biomarkers for GDM. Although promising, data from current studies are still insufficient to draw firm conclusions and some inconsistencies have emerged. The vast majority of the above mentioned studies restricted blood collection for ncRNA analysis to the late second trimester of gestation, or explored ncRNA expression profile in gestational tissues at term. Taken together, these findings have undeniably given significant contribution to the field by helping understand the physiological and pathological hallmarks and pathways in GDM. However, further research should explore ncRNA patterns earlier in pregnancy, in order to significantly predict GDM and, consequently, prevent poor outcomes. Indeed, the controversial results between studies might be partly explained by a differential expression of ncRNA in different stages of pregnancy. Further evidence from prospective studies, evaluating ncRNA patterns from the very early stage of pregnancy and then in different windows of time until delivery, is therefore mandatory to ascertain the reliability of ncRNAs as independent predictors of GDM. Future research might also consider detecting ncRNA in the amniotic fluid collected by invasive diagnostic procedures, such as amniocentesis, performed early in pregnancy for prenatal diagnosis of aneuploidies. Notably, the amniotic fluid might show distinct patterns of ncRNA expression, which could more strictly reflect the metabolic status of the fetus. 

The identification of tissue and/or cell origin of circulating ncRNAs is another crucial point for further research. Over the last decades, growing interest has emerged in characterizing ncRNAs in tissue-specific vesicles, such as placenta-derived exosomes. Surely, advances in this field might significantly contribute to dissect the crosstalk between tissues and biological fluids both in physiological and in pathological contexts.

Discrepancies between studies might also be attributed to the heterogeneity of the enrolled populations or to critical differences related to the analytical methods employed for ncRNA expression profiling. An important issue that makes circulating ncRNA analysis particularly challenging is the choice between plasma and serum samples. Detecting these molecules in plasma avoids bias linked to coagulation. Indeed, the latter process promotes the release of ncRNA from activated platelets [153].

As for analytical methods, it would be of utmost importance in future studies to analyse ncRNAs through absolute quantification and RNA identification. In this attempt, it would be very useful to apply standardized analytical techniques for RNA extraction, such as automated extraction methods, which should be always preferred to manual techniques, in order to reduce sample variability related to manual and technical reasons. In addition, a crucial advance in analytical methodologies can be obtained through performing highly multiplexed single molecule counting. Another relatively new technique but with a great potential is the droplet-digital PCR (ddPCR), which overcomes qPCR in terms of performance and accuracy and does not need replicate samples or reference genes for normalization, giving an absolute quantification of the molecules of interest with an extremely high intra- and inter-assay reproducibility, with the potential to be used both in research and diagnostics [154].

Finally, there are hints that an adverse fetal environment might have a significant impact on the risk of developing T2D and CVD in adulthood. However, the underlying molecular mechanisms remains not yet entirely defined. It is believed that fetal exposure to a hostile environment in GDM pregnancy might induce modifications in ncRNA expression profile, therefore promoting changes in gene expression in the offspring. Further research is needed in the field of epigenetics to unravel the complex potential mechanisms and the involvement of ncRNAs in the fetal programming of T2D and CVD. 

## Figures and Tables

**Table 2:** Studies that evaluated the role and biological functions of lncRNAs in GDM.

Study	Groups	Stage of Pregnancy	Source	lncRNA	Target miRNA	Target Pathway	Role/Biological Function
Zhang et al. [109]	50 GDM 47 CTRL	36th–40th week	Serum	MALAT1 (↑)	-	-	Circulating biomarker of GDM
Wang et al. [110]	13 GDM 15 PE 13 CTRL	Term	Placenta	PVT1 (↓)	miR-10-5p miR-423-5p miR-374b-5p miR-378b miR-150-5p miR-194-5p miR-3184	PI3K/AKT	Trophoblast dysfunction (↓ proliferation and migration)
Ye et al. [111]	16 GDM 18 CTRL	Term	HUVECs	MEG3 (↑)	miR-370-3p	PI3K/AKT	Endothelial dysfunction (↓ tube formation and migration)
Shi et al. [112]	30 GDM-M30 CTRL-M	Term	Plasma (HUVECs)	ENST00000552367 (↑)	-	TADG51/IGF1	↓ Apoptosis
Lu et al. [113]	150 GDM-M 150 CTRL-M 150 CTRL 150 Np	Early pregnancy	Plasma	XLOC_014172 (↑) RP11-230G5.2 (↑)	-	-	Circulating biomarkers of macrosomia in GDM

GDM: gestational diabetes mellitus; CTRL: control subjects; PE: Pre-eclampsia; PVT1: plasmacytoma variant translocation 1; PI3K/AKT: phosphatidylinositol 3-kinase/protein kinase B; MEG3: maternally-expressed gene 3; HUVECs: human umbilical vein endothelial cells (HUVECs); TADG51: T cell death-associated gene 51; IGF1: insulin-like growth factor 1; M: Macrosomia; Np: non-pregnant.
